# Development of human cell-expressed tag-free rhMFG-E8 as a radiation mitigator and a therapeutic for acute kidney injury

**DOI:** 10.21203/rs.3.rs-2809755/v1

**Published:** 2023-05-15

**Authors:** Wayne Chaung, Fangming Zhang, Gaifeng Ma, HaoTing Yen, Asha Jacob, Max Brenner, Ping Wang

**Affiliations:** 1 TheraSource LLC, 350 Community Drive, Manhasset, NY; 2 Center for Immunology and Inflammation, Feinstein Institutes for Medical Research, Manhasset, NY; 3 Departments of Surgery and Molecular Medicine, Zucker School of Medicine, Hempstead, NY

**Keywords:** rhMFG-E8, GI-ARS, medical countermeasure, nuclear terrorism, acute kidney injury

## Abstract

**Background:**

Human milk fat globule epidermal growth factor-factor VIII (MFG-E8) functions as a bridging molecule to promote the removal of dying cells by professional phagocytes. *E. coli*-expressed histidine-tagged recombinant human MFG-E8 (rhMFG-E8) is protective in various disease conditions. However, due to improper recombinant protein glycosylation, misfolding and possible antigenicity, *E. coli*-expressed histidine-tagged rhMFG-E8 is unsuitable for human therapy. Therefore, we hypothesize that human cell-expressed, tag-free rhMFG-E8 can be developed as a safe and effective novel biologic to treat inflammatory diseases such as radiation injury and acute kidney injury (AKI).

**Methods:**

We produced a new tag-free rhMFG-E8 protein by cloning the human MFG-E8 full-length coding sequence without any fusion tag into a mammalian vector and expressed it in HEK293-derived cells. The construct includes the leader sequence of cystatin S to maximize secretion of rhMFG-E8 into the culture medium. After purification and confirmation of the protein identity, we first evaluated its biological activity *in vitro*. We then determined its efficacy *in vivo* utilizing two experimental rodent models of organ injury: partial body irradiation (PBI) and ischemia/reperfusion-induced AKI.

**Results:**

HEK293 cell supernatant containing tag-free rhMFG-E8 protein was concentrated, purified, and rhMFG-E8 was verified by SDS-PAGE analysis and mass spectrometry. The biological activity of human cell-expressed tag-free rhMFG-E8 was superior to that of *E. coli*-expressed His-tagged rhMFG-E8. Toxicity, stability, and pharmacokinetic studies indicate that tag-free rhMFG-E8 is safe, highly stable after lyophilization and long-term storage, and with an adequate half-life for therapeutic applications. In the PBI model, a dose-dependent improvement of the 30-day survival rate was observed after tag-free rhMFG-E8 treatment with a 30-day survival of 89%, which was significantly higher than the 25% survival in the vehicle group. The dose modification factor (DMF) of tag-free rhMFG-E8 was 1.073. Tag-free rhMFG-E8 also attenuated gastrointestinal damage after PBI. In the model of AKI, tag-free rhMFG-E8 treatment significantly attenuated kidney injury and inflammation, and improved the 10-day survival.

**Conclusion:**

Our new human cell-expressed tag-free rhMFG-E8 can be further developed as a safe and effective therapy to treat victims of severe acute radiation injury and patients with acute kidney injury.

## BACKGROUND

Human milk fat globule epidermal growth factor-factor VIII (MFG-E8) is a 387-amino acid glycoprotein also known as lactadherin. It was first identified as an indispensable and major component of the milk fat globule in the lactating mammary glands [1]. Since its discovery, MFG-E8 has shown to be expressed in various cell types including mammary epithelial cells, keratinocytes, dendritic cells, glial cells, splenocytes, monocytes, peritoneal macrophages, and lamina propria macrophages in the small intestine and the colon [2–4]. Human MFG-E8 contains one epidermal growth factor (EGF)-like repeat at the N-terminal and two discoidin domains at the C-terminal. MFG-E8 received its name based on the sequence similarity of the EGF-like repeat to those of EGF and of the discoidin domains to those of the blood coagulation factor V/VIII segments [5]. Its N-terminal EGF-like domain contains an RGD (Arg-Gly-Asp) motif that binds α_v_β_3_ and α_v_β_5_ integrins on the surface of macrophages while the C-terminal, factor VIII-like discoidin domain binds phosphatidylserine (PS) on the surface of apoptotic and other dying cells. Thus MFG-E8 functions as a bridging molecule to promote removal of unwanted cells by professional phagocytes [5, 6]. MFG-E8 deficient mice display inflammation and autoimmunity and have glomerulonephritis due to defects in apoptotic cell engulfment by phagocytes [7]. Since MFG-E8 promotes clearance of apoptotic cells, we reasoned that exogenous administration of MFG-E8 could prevent the exaggerated inflammatory responses seen in acute inflammation such as sepsis [8, 9]. As such, we produced polyhistidine (His)-tagged recombinant human (rh)MFG-E8 using an *E. coli* expression system and conducted proof-of-concept studies in various experimental rodent models of acute inflammation including acute radiation syndrome (ARS) and acute kidney injury (AKI) [10–15].

Acute radiation syndrome is a severe form of radiation injury that occurs after non-medical exposure to high doses of penetrating radiation within a relatively short period of time [16–18]. Exposure to elevated doses of radiation may happen after a natural disaster or an accident in a nuclear facility, or the detonation of a radioactive device or nuclear bomb due to war or international terrorism. ARS can result in three overlapping and progressively severe syndromes. The stem and progenitor cells that regenerate blood cells, and the mucosal lining of the digestive system have elevated mitotic rates, which make them particularly susceptible to damage by ionizing radiation[18]. Exposures to above 1-2 Gy cause the hematopoietic syndrome (H-ARS) characterized by neutropenia resulting in life-threatening infections along with thrombocytopenia leading to spontaneous bleeding. Exposures to above 6-8 Gy cause the gastrointestinal syndrome (GI-ARS) characterized by vomiting, severe diarrhea, structural damage to the gut, and translocation of bacteria from the gut to the circulation. The neurovascular syndrome (NV-ARS) happens after the exposure to extremely elevated doses of radiation (>30 Gy) and invariably progresses to death within days. Several growth factors have been FDA-approved as medical countermeasures (MCMs) to treat patients with H-ARS: Neupogen (filgrastim), Neulasta (pegfilgrastim), Leukine (sargramostim), NPLATE (romiplostim), and Udenyaca (pegfilgrastim-cbqv). No FDA-approved radiation mitigators, however, appear to be effective against GI-ARS. Therefore, there is an urgent unmet need for an effective MCM for GI-ARS. In a rodent model of total body irradiation (TBI), we have previously shown that MFG-E8 levels in the circulation and its mRNA expression in the intestine were significantly reduced after TBI, and that reconstitution of the MFG-E8 deficit with exogenous *E. coli*-expressed His-tagged rhMFG-E8 preserved intestinal histological architecture leading to improved survival after TBI [11]. These results strongly support developing rhMFG-E8 as a biologic mitigator for GI-ARS.

Acute kidney injury (AKI) often results from renal ischemia reperfusion (I/R) during surgical procedures including cardiac or aortic surgeries, tumor resection, renal transplantation, and trauma [19, 20]. In the initial ischemic phase of renal I/R, the hypoxic insult causes cell necrosis whereas during reperfusion, influx of activated neutrophils and macrophages occur leading to secretion of cytokines, proteases, and reactive oxygen species. These events further damage renal tubular epithelial cells and peritubular capillary endothelial cells leading to AKI [21–24]. Although a few pharmacological strategies advanced to clinical trials [25–28], none has been completely effective for AKI patients. Therefore, there is a need for a more effective treatment for AKI. We have previously shown in a mouse model of AKI using renal I/R that MFG-E8 mRNA expression and protein levels in the kidneys dropped by 45% after AKI. Treatment with rmMFG-E8 significantly protected the mice from AKI and had normal levels of blood urea nitrogen (BUN) and creatinine. rmMFG-E8 treatment decreased proinflammatory cytokines, neutrophil infiltration, histological tubular damage, and the number of apoptotic cells present in the kidneys[29]. These preclinical studies support in developing rhMFG-E8 as an effective therapeutic for both GI-ARS and AKI. Glycosylation is thought to enhance the amphipathic properties of MFG-E8 afforded by its membrane binding discoidin domains and its hydrophilic N-terminal tail [30]. However, *E. coli* lacks the cellular machinery to adequately conduct many critical post-translational modifications, including glycosylation and phosphorylation [31]. Consequently, recombinant protein misfolding and aggregation in the form of inclusion bodies are common in prokaryotic expression systems [32]. Moreover, His-tagging increases the immunogenicity of recombinant proteins [33]. Together, inadequate glycosylation, protein misfolding, and potential immunogenicity render *E. coli*-expressed His-tagged rhMFG-E8 unsuitable to be used therapeutically in humans. To circumvent these limitations, we set out to produce a tag-free, normally glycosylated rhMFG-E8 to be further developed as a biologic treatment for radiation injury and AKI. Towards this objective, we cloned the full-length coding sequence of human MFG-E8 devoid of any fusion protein tag into a mammalian vector and expressed in a human cell line. After purification and confirmation of the protein identity, we evaluated its safety and biological activity *in vitro and in vivo*. To test its biological activity *in vitro*, we used a novel PS binding assay and a cell adhesion assay. To test the efficacy *in vivo*, we utilized rodent models of partial body irradiation (PBI) and I/R-induced acute kidney injury (AKI).

## MATERIALS AND METHODS

### Tag-free rhMFG-E8 expression and purification from human cells

The full-length coding sequence of human MFG-E8 (NM_005928.2; Leu24-Cys387), with no fusion tag but addition of the 23 amino acid leader sequence of cystatin S to maximize secretion, was cloned into a mammalian vector and expressed in Expi293F^™^ cells (Life Technologies), which are derived from the human embryonic kidney (HEK) 293 cell line. The human cell-expressed tag-free rhMFG-E8 protein secreted into the culture medium was purified by cation exchange using fast protein liquid chromatography (FPLC) and eluted protein fractions were monitored at 280-nm absorbance and collected. The elution fractions of the target protein were run on SDS-PAGE, detected by Coomassie blue stain for purity confirmation. The gel band containing the target protein was then excised and subjected to MALDI-TOF mass spectrometry for further verification.

### Biological activity assays for human cell-expressed tag-free rhMFG-E8

We developed two quality control assays and one of which is to verify the tag-free rhMFG-E8’s ability to bind to PS on the surface of dying cells (PS binding assay) and another is the tag-free rhMFG-E8 to bind to α_v_β_3_ integrin on the membrane of phagocytes (cell adhesion assay). Briefly, for PS binding assay, we first coated 96-well plate with 100 μl of 3 μg L-a-phosphatidylserine/mL methanol (PS, Avanti Polar Lipids, Inc.) in a laminar flow hood overnight. After washing the plate 3 times with PBS containing 0.05% Tween-20 (PBS-T), the wells were blocked with 2% BSA in PBS for 2 h. After washing again, 100 μl each of serially diluted purified tag-free rhMFG-E8 protein or commercially available mammalian cell-expressed His-tagged rhMFG-E8 (R&D Systems, cat: 2767-MF) was added to PS-coated wells and the plate was incubated for 1 h at room temperature on a rotator. After the washes, 100 μl each of 0.5 μg/ml human MFG-E8 primary antibody (R&D Systems, cat: MAB27671) was added to each well and the plate incubated for 1 h. After the wash, 100 μl each of 1:10,000 dilution of the secondary antibody, goat antimouse IgG-HRP (Southern Biotech, cat: 1012-05) was added to each well and incubated for 1 h. After washing, 100 μl TMB substrate reagent (BD, Cat: 555214) was added to each well and waited for 30 min. Then, 50 μL 2N H_2_SO_4_ stop solution was added to each well. The plate was read at 450 nm with a plate reader (SynergyNeo2, BioTeck). The OD vs. concentration was plotted to detect the amount of rhMFG-E8 that remained bound to the plate and the slope was calculated. For the cell adhesion assay, we determined the ability of rhMFG-E8 to bind α_v_β_3_ integrin. Fluorescently labelled mouse vascular endothelial cells (SVEC 4-10, ATCC), characterized by high surface expression of α_v_β_3_ integrin, were added to wells pre-coated with different concentrations of rhMFG-E8. After washes, the plate was read using a fluorescence plate reader (SynergyNeo2, BioTeck). The percentage of cells that remained bound to the plate was quantified.

### Experimental animals

Sprague Dawley rats (275-350 g) were obtained from Charles River Laboratories. C57BL6 mice were purchased from Jackson Laboratory. Both were housed in a temperature-controlled room with a 12-h light/dark cycle and fed a standard Purina mouse chow diet. Both rats and mice were acclimated to the environment for 5 to 7 days. Age-matched (12-16 weeks for rats and 8-12 weeks for mice) healthy animals were used as controls for the experiments. All experiments involving live animals were approved by the Institutional Animal Care and Use Committee of the Feinstein Institutes for Medical Research and were performed in accordance with the National Institutes of Health and the Guide for the Care and Use of Laboratory Animals.

### Toxicity and stability assessment of human cell-expressed tag-free rhMFG-E8

Non-anesthetized adult male and female healthy mice received a single subcutaneous (*sc*) injection of tag-free rhMFG-E8 at various doses and the mice were monitored daily for 28 days and body weight recorded. Additional mice, untreated or treated with a single *sc* injection of up to 2.0 mg/kg BW tag-free rhMFG-E8, were followed for 1, 7, or 28 days, when blood was collected for further analysis. To assess the stability of the protein after long term storage, tag-free rhMFG-E8 was lyophilized and stored at −20°C. Every four months over a total period of 2 years, the lyophilized protein was reconstituted with pure water and subjected to PS binding and cell adhesion assays to evaluate the stability of its biological activity. MFG-E8 protein purchased from R&D was used each time as a control for assay.

### Determination of pharmacokinetic properties of human cell-expressed tag-free rhMFG-E8

To allow for repeated collection of sufficient amounts of blood, pharmacokinetic studies were performed in Sprague Dawley rats. Rats were intravenously injected with 50 μg of tag-free glycosylated rhMFG-E8. Blood samples (100 μl per time point) were collected at 0, 3, 6, 9, 12 and 15 min, then every 15 min for the first 60 min, and then every hour for additional 11 h. Equal volume (100 μl) of blood withdrawn from the rat was replenished with normal saline. The total volume of blood collected (1.6 ml) was approximately 8% of the total body blood volume (~20 ml/rat). The levels of the tag-free rhMFG-E8 from rat blood samples were measured by Human MFG-E8 Quantikine ELISA kit (R&D Systems, Cat: MFGE80). There is no cross-reaction between human and rat MFG-E8 in this kit. The data were plotted as serum rhMFG-E8 concentration vs. time. The slopes of the distribution (α) and elimination (β) phases were calculated.

### Mouse model of PBI and treatment with human cell-expressed tag-free rhMFG-E8

Non-anesthetized adult male and female mice were exposed to a PBI dose of 15 gray (Gy) in an X-Ray irradiator (model: X-RAD 320, Pxi, North Branford, CT) at a rate of approximately 1 Gy/min at 320 KV, 12.5 mA, 50 cm SSD. We first exposed the mice to different doses of X-ray using PBI model and determined 15 Gy as the optimal dose to ensure high mortality rate in C57BL/6J mice. During irradiation, mice were briefly restrained and placed in a fitted container with the hind extremities (fibula, tibia and feet) were shielded using lead tubes, thereby protecting approximately 5% of the bone barrow. After PBI, the mice were returned to their cages. At 24 h later, mice were *sc* injected daily for 6 days with normal saline (vehicle) or tag-free rhMFG-E8 (treatment) at various doses. The mice were monitored daily for 30 days, and survival rate recorded.

### Determination of dose modification factor (DMF) of human cell-expressed tag-free rhMFG-E8

Mice were exposed to PBI at staggered doses of 13, 13.5, 14, 14.5, 15 and 16 Gy. At 24 later, they were injected *sc* with either vehicle or 1 μg/kg BW tag-free rhMFG-E8 daily for 6 consecutive days, monitored daily for 30 days and survival recorded. The survival data were plotted as percent mortality vs. PBI dose and DMF was calculated.

### Assessment of gut damage after PBI and treatment with human cell-expressed tag-free rhMFG-E8

Mice exposed to 14.5 Gy PBI were injected *sc* with 1 μg/kg BW tag-free rhMFG-E8 for 3 consecutive days starting at 24 h after PBI. They were then euthanized, and blood and jejunal segments of the gut were harvested for further analysis. The serum levels of citrulline from PBI samples were measured using commercially available enzyme-linked immunosorbent assay (ELISA) kits (Mouse Citrulline ELISA Kit, MyBioSource, cat # MBS2602136). Plasma citrulline has been identified as a biomarker of radiation-induced gut damage [34]. Jejunal segments were paraffin-embedded, sectioned, and stained with H&E. Gut histological damage score for PBI samples was quantified using RIIMS, a detailed 7-category scoring system previously described [11].

### Mouse model of renal I/R and treatment with human cell-expressed tag-free rhMFG-E8

Renal I/R was performed as previously described [29] with modification. Adult male mice underwent anesthesia induction via 2-4% isoflurane inhalation, after which the ventral abdomen was shaved and disinfected with betadine and 70% alcohol. A 1-2 cm midline incision was performed, and the bowels were displaced to expose the kidneys. Microvascular clips were placed at both the left and right renal pedicles for 30 min. After removal of the clips, either normal saline (vehicle) or 20 μg/kg BW tag-free rhMFG-E8 (treatment) were injected intraperitoneally, and the abdomen was closed. Then mice were *sc* injected with 1 ml normal saline as resuscitation fluid and buprenorphine (50 μg/kg BW) as analgesia. After recovery from surgery, mice were returned to their home cages. Sham mice underwent similar procedure except for clamping. At 24 h or 48 h later, serum and kidneys were collected; kidneys were either fixed in 10% formalin (histology) or frozen in liquid nitrogen and stored at −80°C, for later analysis. Additional mice that underwent renal I/R and treatment were monitored twice daily for 10 days and the survival recorded.

### Measurement of serum injury markers and cytokine levels

Whole blood samples were centrifuged at 3,000 x g for 10 min. Serum samples collected were then stored at −80°C until use. Serum levels of AST, ALT, BUN and creatinine were measured using commercially available reagents (Pointe Scientific). Serum levels of proinflammatory cytokines, TNF-α, IL-6 and IL-1β were measured using ELISA kits (BD Biosciences).

### Assessment of kidney cytokine and chemokine mRNA expression

Total RNA isolated from kidney tissues by TRIzol reagent (Invitrogen) was reverse-transcribed to cDNA using reverse-transcriptase (Applied Biosystems). PCR reactions were carried out with 0.08 μM of each forward and reverse primer, cDNA, water and 2X SYBR Green master mix (Applied Biosystems) in a final volume of 20 μl. Amplification was performed in a StepOne Plus real-time PCR machine (Applied Biosystems). Mouse β actin was used as an internal control and the relative mRNA expression was calculated using 2^−ΔΔCt^ method. The following list of primer sequences were used: TNF-α, 5’-AGACCCTCACACTCAGATCATCTTC-3’ (forward), and 5’- TTGCTACGACGTGGGCTACA-3’ (reverse); IL-6, 5’-CCGGAGAGGAGACTTCACAG-3’ (forward) and 5’- CAGAATTGCCATTGCACAAC-3’ (reverse); IL-1β, 5’-CAGGATGAGGACATGAGCACC-3’ (forward), and 5’-CTCGCAGACTCAAACTCCAC- 3’ (reverse); KIM-1, 5’ TGCTGCTACTGCTCCTTGTG 3’ (forward), and 5’ GGGCCACTGGTACTCATTCT 3’ (reverse); IL-18, 5’ GCCTCAAACCTTCCAAATCA 3’ (forward) and 5’ TACAGTGAAGTCGGCCAAAG3’ (reverse); β-actin, 5’-CGTGAAAAGATGACCCAGATCA-3’ (forward), and 3’-TGGTACGACCAGAGGCATACAG-3’ (reverse).

### Histological assessment of renal injury

Kidney tissues were dissected, fixed in formalin, paraffin-embedded, sectioned, and stained with hematoxylin and eosin (H&E). Kidney injury was assessed by measuring severity of tubular necrosis, loss of brush border and cast formation on a scale of 1-5 with the maximum score of 15 [35]. Scores were averaged for each sample over 10 randomly selected fields.

### TUNEL assay

Kidney tissues harvested after renal I/R were sectioned into 5-μm slices and subjected to Terminal deoxynucleotidyl transferase dUTP nick end labeling (TUNEL) assay using a commercially available “In Situ Cell Death Detection kit” according to manufacturer’s instructions. 4’, 6-diamidino-2 phenylindole (DAPI) was used as a nuclear counterstain. TUNEL positive cells were counted using ImageJ software.

### Statistical analysis

Results are expressed as mean ± SEM and compared by using one-way analysis of variance (ANOVA) and Student Newman-Keul’s (SNK) post hoc analysis. Survival rates were analyzed by the Kaplan-Meier estimation and compared using log-rank test. Differences in values were considered significant if P<0.05. Data analyses were conducted using GraphPad statistical program and software (GraphPad).

## RESULTS

### Synthesis and purification of human cell-expressed tag-free rhMFG-E8

**Figure 1.**
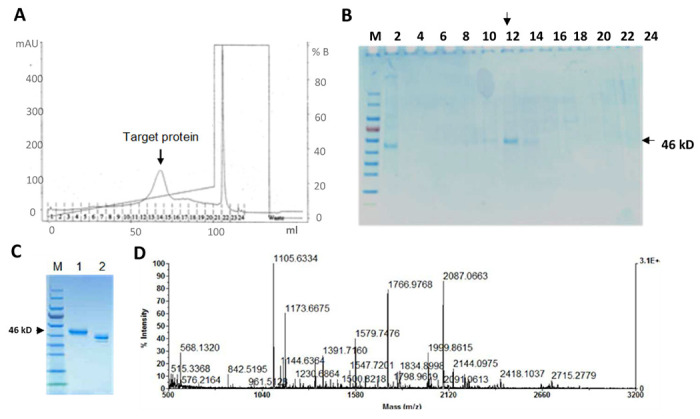
Expression and purification of tag-free rhMFG-E8. Tag-free rhMFG-E8 was expressed in human cells and purified. (**A**) The eluted protein’s 280-nm absorbance was monitored using FPLC and the target protein fraction (arrow) identified. (**B**) Eluted protein fractions from FPLC were confirmed by polyacrylamide gel electrophoresis, and (**C**) fraction #14 was resolved separately in a NuPAGE 4-12% Bis-Tris gel under reducing (Lane 1) and non-reducing (Lane 2) conditions. (**D**) The purified protein was identified as human MFG-E8 by MALDI-TOF mass spec analysis.

Tag-free rhMFG-E8 with 23 aa leader sequence of cystatin S was cloned into a mammalian expression vector, expressed in human HEK cells, and purified with cation exchange using FPLC. The eluted protein fractions were monitored with 280-nm absorbance and a target protein peak was identified (Fig. 1A) and collected. After optimization of the expression procedure, the concentration of the tag-free glycosylated rhMFG-E8 was high enough to be detected directly by SDS-PAGE and fraction #14 contained the majority of the expressed rhMFG-E8 protein, with a concentration of 0.33 μg/μl (Fig. 1B). The purity of this fraction was further verified by SDS-PAGE under reducing and non-reducing conditions (Fig. 1C), and the excised gel band protein was identified as human MFG-E8 using mass spectrometry (Fig. 1D). The purified tag-free rhMFG-E8 using human cell expression system had an endotoxin level below 0.02 EU/μg (data not shown), about two orders of magnitude lower than the industry standard (<1 EU/μg).

### Human cell-expressed tag-free rhMFG-E8 has high and stable biological activity

**Figure 2.**
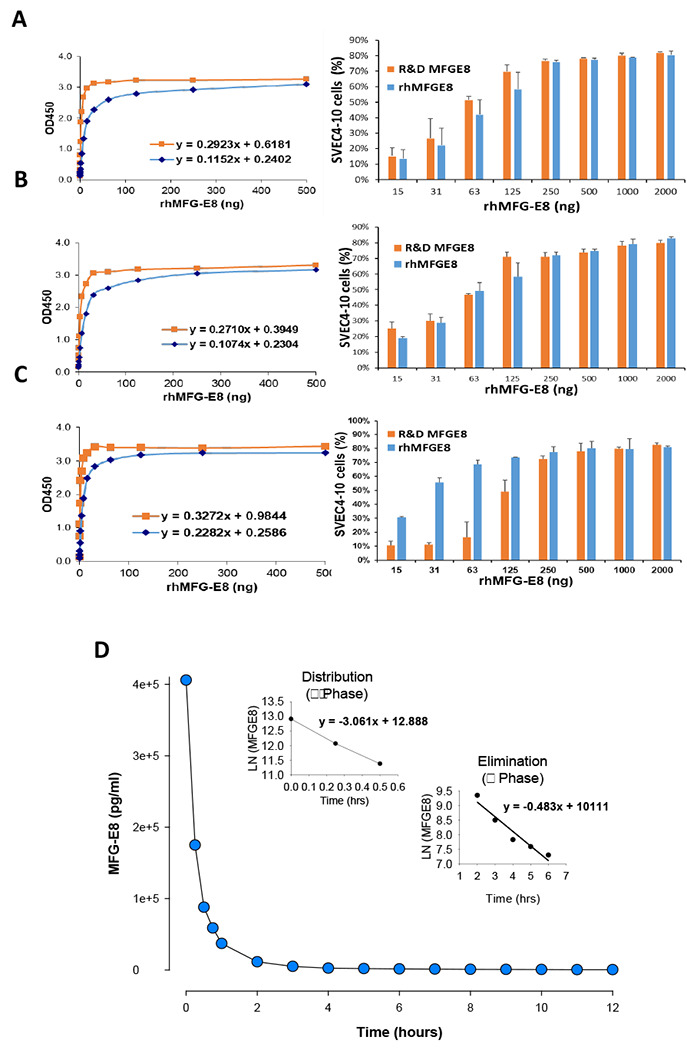
(**A**) *Tag-free rhMFG-E8 has high biological activity*. PS binding (left panel) and SVEC4-10 cell adhesion assay (right panel) of commercial MFG-E8 (R&D MFG-E8) and tag-free rhMFG-E8 (rhMFG-E8). (**B-C**) *Tag-free rhMFG-E8 is highly stable*. PS binding and cell adhesion assays performed at (**B**) twelve months, (**C**) twenty-four months after storage of the tag-free rhMFG-E8 at −20°C. **D**. *Tag-free rhMFG-E8 has suitable pharmacokinetics in healthy mice*. Rats were *iv* injected with tag-free MFG-E8 and blood samples collected at different timepoints. The levels of the tag-free rhMFG-E8 were measured using human MFG-E8 Quantikine ELISA kit. The data were plotted as serum rhMFG-E8 concentration against time points to determine the t_1/2_.

The biological activity of the tag-free rhMFG-E8 was assessed using two methods. We independently verified its ability to bind to both PS and α_v_β_3_ integrin. To determine the PS binding ability, various concentrations of tag-free rhMFG-E8 were added to PS-coated wells and the amount of tag-free rhMFG-E8 that remained bound to the plate was assessed using a chromogenic immunoassay with anti-MFG-E8 antibodies. The PS binding activity of human cell-expressed tag-free rhMFG-E8 was significantly increased than that of *E. coli*-expressed His-tagged rhMFG-E8 (Suppl. Fig. 1A). We compared our human cell-expressed tag-free rhMFG-E8 against commercially available rhMFG-E8 (R&D), which is also mammalian cell-expressed but unsuitable to be used therapeutically because it is His-tagged. As indicated by a steep linear phase slope, tag-free rhMFG-E8 had very comparable PS-binding activity to that of His-tagged commercially available rhMFG-E8 (Fig. 2A, **left panel**). To determine the ability of tag-free rhMFG-E8 to bind to α_v_β_3_ integrin, fluorescently labelled mouse vascular endothelial cells SVEC4-10, which express α_v_β_3_ integrin on their surface, were added to wells pre-coated with different concentrations of tag-free rhMFG-E8. After washing, the percentage of fluorescent cells that remained bound to the plate was quantified. Echoing our PS binding results, the cell adhesion binding activity of human cell-expressed tag-free rhMFG-E8 was also markedly higher than that of *E. coli*-expressed His-tagged rhMFG-E8 (Suppl. Fig. 1B). Tag-free rhMFG-E8 also bound SVEC4-10 cells more strongly and at much lower concentrations than the His-tagged commercially available rhMFG-E8 (Fig. 2A, **right panel**). These results demonstrate that the biological activity of tag-free rhMFG-E8 is far superior to that of *E. coli*-expressed His-tagged rhMFG-E8, and comparable (if not better) to His-tagged commercially available rhMFG-E8. In terms of stability of lyophilized, stored, and then reconstituted tag-free rhMFG-E8, we conducted a total of 12 independent sets of PS binding and cell adhesion assays over a 2-year period. Our results indicated that both PS binding ability and SVEC4-10 cell binding capability of the stored tag-free rhMFG-E8 were highly similar to those of His-tagged commercially available MFG-E8, which benefits from R&D’s excellent industrial quality control assessment (Fig. 2B–C).

### Human cell-expressed tag-free rhMFG-E8 has improved and stable biologic activity, is safe, and has adequate pharmacokinetic properties

To screen for major toxicity, we injected naïve mice with tag-free rhMFG-E8 and analyzed the sera collected at 1-, 7-, and 28-days post-injection for the organ injury biomarkers AST, ALT, LDH, creatinine, BUN, and for the pro-inflammatory cytokines TNF-α and IL-6. All biomarker and cytokine measurements were within normal levels suggesting tag-free rhMFG-E8 treatment of healthy mice causes neither acute nor chronic toxicity (Table 1). We attempted to determine the maximum tolerated dose, but no deaths were observed in mice treated with doses of up to 2 mg/kg BW tag-free rhMFG-E8 and they didn’t undergo any significant changes in body weight. These results showed that very high doses of tag-free rhMFG-E8 (up to 200-fold higher than the highest dose used in this study) produced no measurable toxicity in mice. To assess pharmacokinetics, wild type Sprague Dawley rats were injected with a bolus dose of 50 μg tag-free rhMFG-E8 and serial blood samples collected at different time points were analyzed for the concentration of the exogenous tag-free rhMFG-E8 using an ELISA kit that does not recognize rat MFG-E8. The terminal elimination half-life (t_1/2_) of the tag-free rhMFG-E8 was calculated for each of 4 independent experiments and averaged. Tag-free rhMFG-E8’s average α (distribution) t_1/2_ was 13.59 ± 0.86 min, and its average β (elimination) t_1/2_ was 1.43 ± 0.19 h (Fig. 2D).

These results collectively demonstrated that human cell-expressed tag-free rhMFG-E8 has superior biological activity, is highly stable, safe, and has suitable pharmacokinetic properties to be used as a novel biologic treatment.

### Human cell-expressed tag-free rhMFG-E8 showed dose-related improvement in survival after PBI

**Figure 3.**
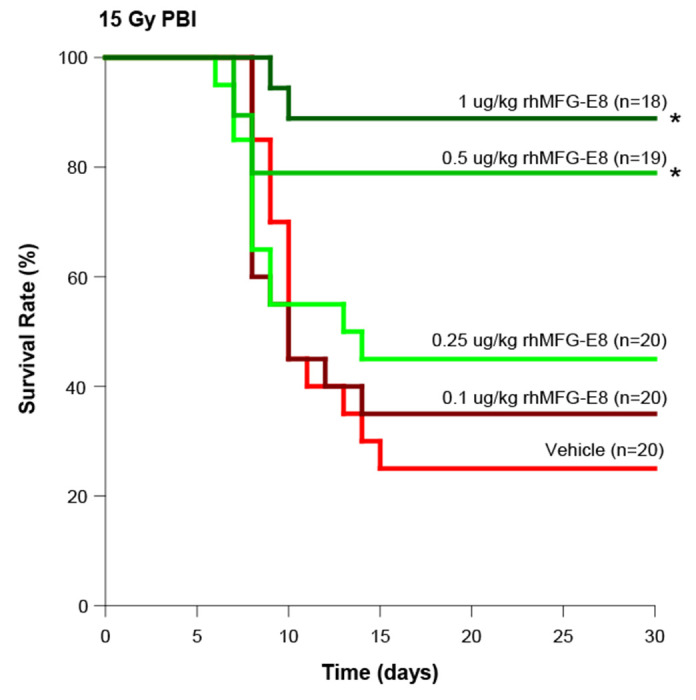
Dose response relationship of tag-free rhMFG-E8. Mice were exposed to PBI at 15 Gy and received sc injections of normal saline (Vehicle) or the indicated various amounts of tag-free rhMFG-E8 (rhMFG-E8) for 6 days, starting at 24 h after PBI. The 30-day survival was recorded. *P < 0.05 vs. Vehicle.

The PBI model was then used to evaluate the effect of tag-free rhMFG-E8 on GI-ARS. We subjected mice to 15-Gy PBI and, at 24 h after irradiation, treated them with either vehicle or varying doses of tag-free rhMFG-E8 to determine the optimal dose of rhMFG-E8 to improve survival. We observed a dose-dependent improvement of the 30-day survival rate with the treatment group (Fig. 3). The highest survival rate reached 89% at 1 μg/kg BW tag-free rhMFG-E8 group, which was significantly higher than the vehicle group at 25%. When administered tag-free rhMFG-E8 at 2 μg/kg BW, it did not show further improvement in survival rate in comparison to the 1 μg/kg BW group. Therefore, 1 μg/kg BW tag-free rhMFG-E8 was the optimal dose to improve survival after PBI.

### Human cell-expressed tag-free rhMFG-E8 produced an effective dose modification factor (DMF) after PBI

**Figure 4.**
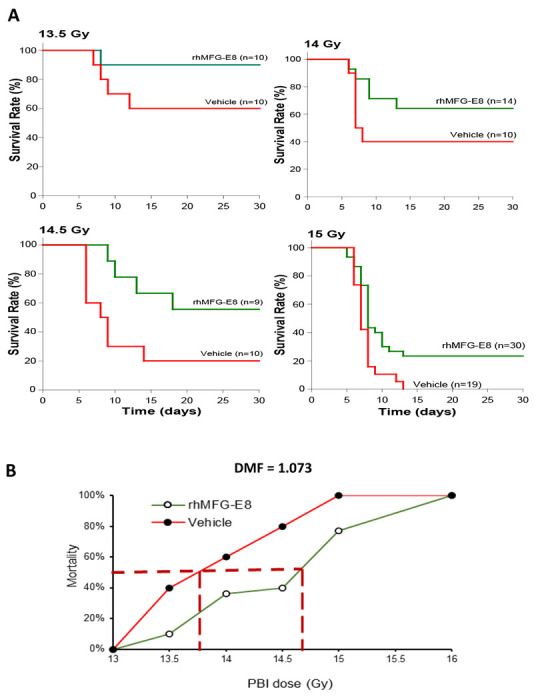
Determination of the *dose modification factor (DMF) in PBI mice*. (**A**) Mice were exposed to PBI at the indicated doses and injected sc with normal saline (Vehicle) or 1 ug/kg tag-free rhMFG-E8 (rhMFG-E8) for 6 days starting at 24 h post-PBI, observed for 30 days and the survival recorded. (**B**) Percent mortality was plotted against each irradiation dose for the 30-day survival. Horizontal dotted red line indicates the mortality rate at 50% and the duplicate vertical dotted lines indicate PBI dose corresponding to 50% mortality for the different groups.

The DMF, also called dose reduction factor, has been considered the best effectiveness index for radiation MCM and is defined as a ratio between the dose of irradiation required for a given effect of a drug-treated group and that of the vehicle-treated group. DMF is essential to label a drug as a radiation MCM. To determine the DMF, a “staggered-dose of radiation” design provides a better evaluation of variability in lethality across the radiation doses than “same-dose of radiation” comparisons. Using the staggered-dose approach, mice were irradiated at 13, 13.5, 14, 14.5, 15 and 16 Gy PBI and 24 h later, they were treated with either normal saline (vehicle) or 1 μg/kg BW tag-free rhMFG-E8 *sc* for 6-days. The 30-day survival rates were recorded (Fig. 4A). Vehicle and rhMFG-E8 treated mice had no mortality after 13-Gy PBI, and 100% mortality was observed after 16-Gy PBI. The survival results were then re-plotted as mortality percentage against each irradiation dose to determine the DMF (Fig. 4B). The LD_50/30_ of the vehicle group was projected as 13.7 Gy, while it was extrapolated as 14.7 Gy for the tag-free rhMFG-E8 treated group. Thus, the LD_50/30_ DMF of tag-free rhMFG-E8 at 1 μg/kg BW was calculated as 1.073 (Fig. 4B) indicating that the treatment increased the radiation dose needed to kill 50% of the mice by 1 Gy, which is a critical benchmark requirement for candidate GI-ARS MCMs [36].

### Human cell-expressed tag-free rhMFG-E8 restored plasma citrulline levels and improved gut histology after PBI

**Figure 5.**
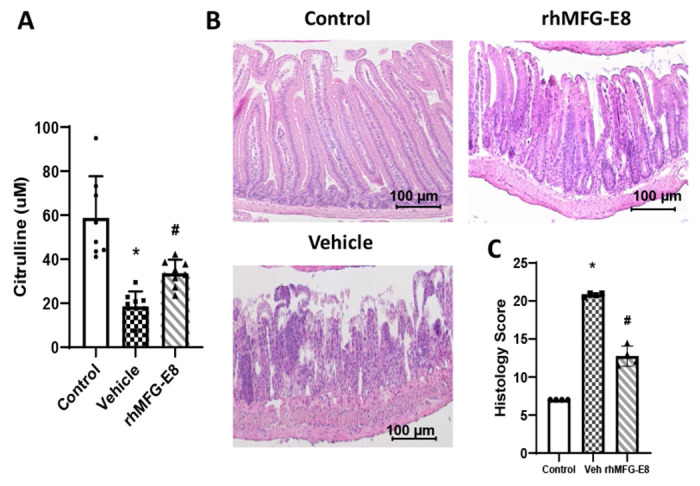
Tag-free rhMFG-E8 increased plasma citrulline and improved gut histology in PBI mice. (**A**) Mice were exposed to 14.5 Gy PBI and injected sc with normal saline (Vehicle) or 1 μg/kg tag-free rhMFG-E8 (rhMFG-E8) starting at 24 h post-PBI and blood samples were collected at 3-days after PBI. Serum levels of citrulline were measured by Mouse Citrulline ELISA Kit. Mice that were not exposed to irradiation was used as control group. (**B**) Representative images of gut histological samples from Day-3 after PBI are shown. (**C**) The histological damage score quantified using RIIMS are shown. Data are represented as mean ± SE (n=8/group) and analyzed using one way ANOVA and Student Newman-Keuls test. *P<0.05 versus control; #P<0.05 versus Vehicle.

Citrulline is a nitrogen end-product of glutamine metabolism in small-bowel enterocytes, and plasma citrulline has been identified as a biomarker of radiation-induced gut damage[34]. To assess gut damage, mice were subjected to 14.5-Gy PBI and at 24 h later, they were treated daily with 1 μg/kg BW tag-free rhMFG-E8. Our data showed that the serum levels of citrulline were decreased in vehicle group as early as 3-days after PBI whereas tag-free rhMFG-E8 treatment group showed a significant increase in citrulline levels (Fig. 5A). These results indicate that improved enterocyte activity could be detected as early as after only two administrations of tag-free rhMFG-E8. We also assessed rhMFG-E8’s effects on the intestinal histological architecture. Regenerative crypts appeared very early following radiation injury in treated mice. Treatment with tag-free rhMFG-E8 in PBI mice attenuated histological damage and further confirmed the protective effect of tag-free rhMFG-E8 on radiation-induced gut damage (Fig. 5B). We further quantified the histological changes using RIIMS and showed that tag-free rhMFG-E8 treatment significantly attenuated gut damage (Fig. 5C). These results demonstrated tag-free rhMFG-E8’s efficacy *in vivo*, and support further developing it as a biological MCM for GI-ARS.

### Human cell-expressed tag-free rhMFG-E8 improved renal function and reduced systemic inflammation in AKI

**Figure 6.**
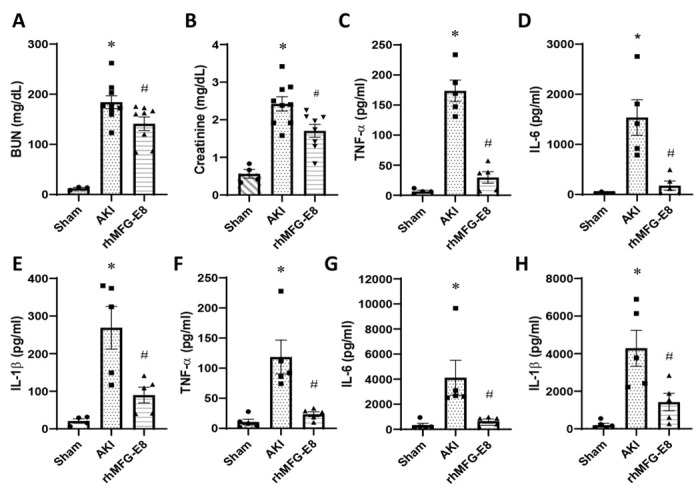
Tag-free rhMFG-E8 reduced kidney injury markers and cytokines after AKI. Mice underwent AKI and treated *ip* with tag-free rhMFG-E8 (rhMFG-E8) and serum levels of (**A**) BUN, (**B**) Creatinine at 48 h after AKI were measured. Serum levels of 24 h (**C-E**) and 48 h (**F-H**) were analyzed for (**C, F**) TNF-α, (**D, G**) IL-6, and (**E, H**) IL-1β. Data are represented as mean ± SE (n=8/group) and analyzed using one way ANOVA and Student Newman-Keuls test. *P<0.05 versus Sham; #P<0.05 versus AKI.

To evaluate tag-free rhMFG-E8 effects on renal function recovery after AKI, mice were subjected to bilateral renal I/R and treated with either vehicle or 20 μg/kg BW tag-free rhMFG-E8. At 24 h, BUN and creatinine serum levels were increased in the vehicle group but were reduced, albeit not yet significantly, in mice treated with tag-free rhMFG-E8. At 48 h after AKI, BUN and creatinine levels were markedly increased in the vehicle group while the levels were significantly decreased by 23% and 30%, respectively, in mice treated with tag-free rhMFG-E8 (Fig. 6A–B). To assess tag-free rhMFG-E8 effects on inflammation after AKI, pro-inflammatory cytokine levels were measured in serum samples from different groups. At 24 h after AKI, serum levels of TNF-α, IL-6 and IL-1β were increased in the vehicle group while significantly decreased by 58%, 64% and 50% in mice treated with tag-free rhMFG-E8 (Fig. 6C–E). At 48 h after AKI, serum TNF-α, IL-6 and IL-1β were increased in the vehicle group whereas these levels were significantly reduced after tag-free rhMFG-E8 treatment by 80%, 84% and 67%, respectively (Fig. 6F–H). These results demonstrated that tag-free rhMFG-E8 improves renal function and reduces systemic inflammation in AKI.

### Human cell-expressed tag-free rhMFG-E8 decreases renal mRNA levels of pro-inflammatory mediators and renal injury biomarkers

**Figure 7.**
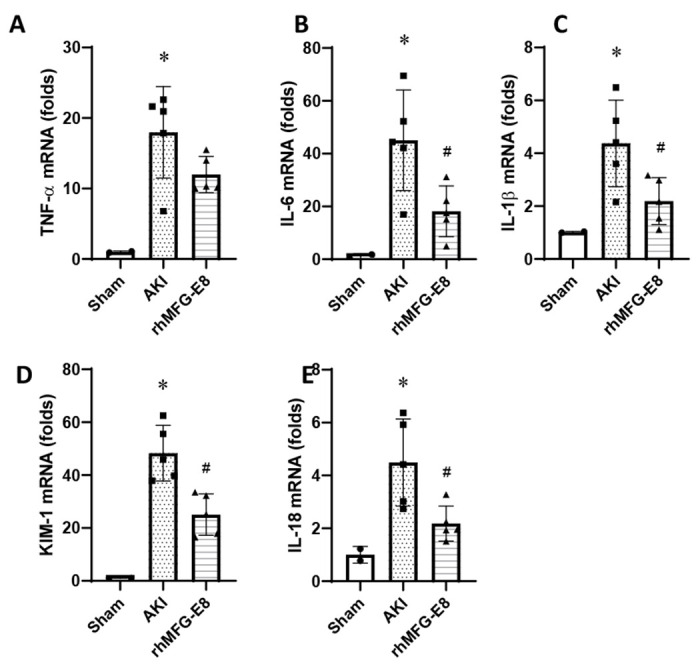
Tag-free rhMFG-E8 reduced kidney cytokines and chemokines after AKI. Kidney tissues from different groups collected at 24 h after AKI were analyzed for mRNA expressions of (**A**) TNF-α, (**B**) IL-6, (**C**) IL-1β, (**D**) KIM-1, (**E**) IL-18 by RT-qPCR. Data are represented as mean ± SE (n=5/group) and analyzed using one way ANOVA and Student Newman-Keuls test. *P<0.05 versus Sham; #P<0.05 versus AKI.

To assess renal inflammation and injury, mRNA levels of pro-inflammatory cytokines and chemokines were measured in the kidneys from different groups 24 h after AKI. Kidney mRNA expression of TNF-α, IL-6 and IL-1β was increased after AKI while tag-free rhMFG-E8 decreased these levels by 34%, 60% and 50%, respectively (Fig. 7A–C). Kidney KIM-1 and IL-18 mRNA levels were increased after AKI while these levels were decreased by 48% and 52%, respectively with tag-free rhMFG-E8 treatment (Fig. 7D–E). These findings showed that tag-free rhMFG-E8 reduces renal inflammation and injury after renal I/R.

### Human cell-expressed tag-free rhMFG-E8 attenuated renal injury and apoptosis after AKI

**Figure 8.**
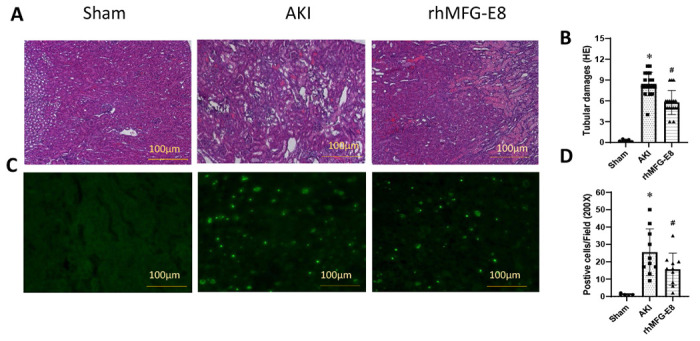
Tag-free rhMFG-E8 improved renal histology and reduced apoptosis after AKI. Mice underwent AKI and treated *ip* with tag-free rhMFG-E8 (rhMFG-E8) and kidney tissues were harvested at 48 h after AKI. Tissue sections were stained with **(A)** H&E for histology and **(B)** tubular damage score assessed. Sections were also stained with **(C)** TUNEL to assess apoptosis and **(D)** positive cells/field were quantified. Data are represented as mean ± SE (n=8/group) and analyzed using one way ANOVA and Student Newman-Keuls test. *P<0.05 versus Sham; #P<0.05 versus AKI.

To measure renal injury at the histological level, kidney tissues were sectioned and stained with H&E and tubular damage score was assessed. At 48 h after AKI, vehicle treated mice showed severe disruptions in the histological architecture compared to sham mice (Fig. 8A). The vehicle group had a kidney injury score of 8.4 but in mice treated with tag-free rhMFG-E8 had a significant 31% decrease with a score of 5.8 (Fig. 8B). To evaluate apoptosis, histological sections were stained with TUNEL. The apoptotic cells identified by green fluorescence were clearly visible in the vehicle group (Fig. 8C). The average number of TUNEL positive cells per high magnification field in the vehicle group was 25.5 ± 4.2 (Mean ± SE), whereas in the tag-free rhMFG-E8 treatment group, the number was significantly decreased by 38% (Fig. 8D). These results further supported amelioration of AKI in mice treated with tag-free rhMFG-E8.

### Human cell-expressed tag-free rhMFG-E8 improved survival in AKI

**Figure 9.**
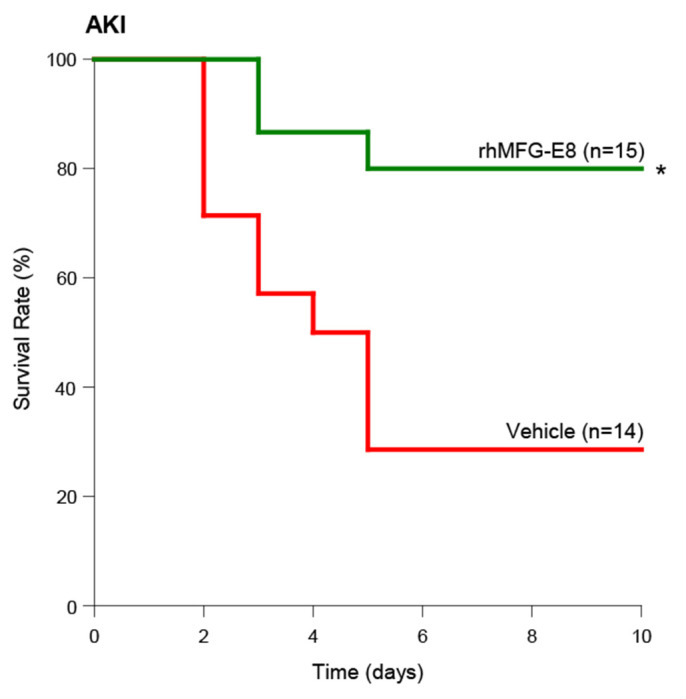
Tag-free rhMFG-E8 improved survival after AKI. Mice underwent AKI and treated *ip* with tag-free rhMFG-E8 and observed for 10 days and survival recorded. The survival rate was analyzed by Kaplan-Meier survival analysis and compared by the log-rank test. *P<0.05 versus AKI.

To determine the long-term beneficial effect of tag-free rhMFG-E8 in AKI, mice subjected to renal I/R and treated with normal saline (vehicle) or 20 μg/kg tag-free rhMFG-E8 (rhMFG-E8) and were monitored for survival for 10 days. Administration of tag-free rhMFG-E8 significantly increased the survival rate from 29% in the vehicle group to 73% in treatment group (Fig. 9). Collectively, our renal I/R studies also demonstrated tag-free rhMFG-E8’s efficacy *in vivo*, and support further developing it as a biological therapeutic for patients with AKI.

## DISCUSSION

MFG-E8, also known as lactadherin in humans, is a glycoprotein secreted by activated phagocytes that bridges the phosphatidylserine (PS) residues on the surface of dying (mostly apoptotic, but also autophagic, necrotic, necroptotic, pyroptotic) cells with α_v_β_3_ and α_v_β_5_ integrins on the surface of macrophages and dendritic cells [4, 5, 37, 38]. MFG-E8 promotes the uptake and clearance of dying cells and prevents local and systemic immune activation and end-organ damage during sepsis, ischemia/reperfusion injury and shock. We have previously demonstrated the beneficial effect of bacterially expressed His-tagged rhMFG-E8 in various organ injury conditions [11–15, 39]. However, unlike eukaryotic expression systems, *E. coli* lack the ability to produce complete and proper and post-translational modifications such as glycan glycosylation, which are important for the adequate protein folding and biological activity of glycoproteins such as MFG-E8. Moreover, *E. coli* expressed proteins run the risk of carry-over of endotoxin and other toxic bacterial components. While the presence of a His-tag facilitates recombinant protein recovery and purification, at the same time it can render proteins immunogenic and thus unsuitable for drug development. To circumvent these undesirable effects, we used a human cell expression system (HEK293 cells) to produce tag-free rhMFG-E8, which we then purified, authenticated, quality-controlled for biological activity *in vitro*, and demonstrated to be effective *in vivo* using two distinct preclinical models of human disease.

The biological integrity of the tag-free rhMFG-E8 was examined *in vitro* by a PS binding assay, which we have developed and standardized in house, as well as by the classical cell adhesion assay. It was evident from its steep linear phase slope that the tag-free rhMFG-E8 was significantly better than *E. coli*-expressed his-tagged rMFG-E8, and had comparable PS binding activity to also mammalian cell-expressed but His-tagged commercially available rhMFG-E8. Tag-free rhMFG-E8 also bound significantly more strongly to SVEC4-10 cells than *E. coli*-expressed his-tagged rhMFG-E8, and equally or more strongly than commercial rhMFG-E8 in all different dilutions, indicating strong ability of the tag-free rhMFG-E8 to bind to the integrin receptors α_v_β_3_ and α_v_β_5_. These results mean that tag-free rhMFG-E8 can be lyophilized into powder form, stored for long periods of time, and reconstituted all the while preserving its full biological function. Moreover, mice administered up to 2 mg/kg BW tag-free rhMFG-E8 (200-fold the highest therapeutic dose in our study) showed no detectable toxicity or inflammation and no significant changes in body weight for up to 28 days after the administration of tag-free rhMFG-E8, indicating lack of toxicity. Nevertheless, additional experiments are required for assessing longer term safety. Additionally, tag-free rhMFG-E8 protein had a relatively short half-life that is adequate for commonly used drug administration regimens, and suggest it is very unlikely that rhMFG-E8 will accumulate in the body to cause adverse effects. Finally, the endotoxin level in tag-free rhMFG-E8 was substantially lower than the industry standard. Together, these observations clearly demonstrate the biological activity, safety, and stability of the tag-free rhMFG-E8 *in vitro*.

To evaluate the biological activity of the human cell-expressed tag-free rhMFG-E8 *in vivo*, an experimental mouse model of PBI was utilized. PBI is accomplished by sparing the bone marrow either extensively (40%) or minimally (5%). Our study was conducted using the 5% bone marrow sparing model. This model was chosen because it is an established model in primates to assess MCM effects on the major radiation syndromes (H-ARS, GI-ARS, DEARE) [36]. In the current study of 15 Gy PBI, survival rate significantly improved from 25% in the vehicle to 89% with 1 μg/kg BW treatment at LD_50/30_ demonstrating the efficiency of the tag-free rhMFG-E8 in ameliorating PBI-induced ARS. Animal survival after TBI can increase from 37% to 87% with a small increment in the radiation dose [40]. Indeed, we observed 100% survival with 13 Gy and 100% mortality with 15 Gy in our PBI model. Therefore, the effectiveness of the 1 μg/kg BW tag-free rhMFG-E8 was assessed using full dose response curves from 13 Gy to 16 Gy in increments of 0.5 Gy. we measured effectiveness using the standardized method of DMF, i.e., the radiation dose required for a given effect (in our case, for LD_50/30_) in treated animals divided by the radiation dose leading to the same effect in the control group [36]. Treatment with tag-free rhMFG-E8 caused a 1-Gy shift in the radiation LD_50/30_ thus fulfilling part of the FDA’s “animal rule” efficacy requirement to approve candidate MCMs for GI-ARS [36].

Another criterium for assessing a candidate MCM is that it should ameliorate the intestinal abnormalities characteristic of GI-ARS such as epithelial denudation and crypt loss. Thus, in mice irradiated with PBI, we also assessed the beneficial effects of tag-free rhMFG-E8 on the plasma levels of citrulline and the histological architecture of the small intestine. Citrulline is an epithelial tissue-specific nitrogen end product of glutamine metabolism in small-bowel enterocytes. Since a lower plasma concentration of citrulline correlates with decreased enterocyte mass/integrity, its circulating levels have been used as a GI-ARS severity biomarker [34]. After PBI, we observed a significant decrease in citrulline levels and intestinal histological injury in the vehicle group, but treatment with tag-free rhMFG-E8 significantly ameliorated enterocyte mass and function, as indicated by the partially restored citrulline levels. Control mice subjected to PBI also had loss of intestinal crypts and breakdown of the mucosal barrier, with sloughing of the tips of the villi and denudation of the epithelial cell layer. On the other hand, mice treated with tag-free rhMFG-E8 had significantly lower intestinal histological injury scores. Breakdown of the intestinal mucosal barrier causes electrolyte imbalances and bacterial translocation (including their toxins) through the intestinal wall into the bloodstream, thus predisposing to infection and inflammation. It is pertinent to also note the paucity of abnormal mitotic nuclei in the crypt after treatment with tag-free rhMFG-E8. This positive effect of tag-free rhMFG-E8 on the gut after PBI can also be attributed, at least in part, to MFG-E8’s trophic effects leading to the repair damaged intestinal epithelium and preserved gut homeostasis [4, 41].

In our study, we chose to employ adult C57BL/6J mice because they are the most widely studied strain and species in preclinical studies of radiation injury [36]. Furthermore, C57BL/6 mice exhibit a moderate degree of radiosensitivity compared to other strains [42]. Some studies indicated that radiosensitivity depends on the timing of irradiation [43] and that some anesthetics can act as radiation protectants [44]. Therefore, all mice were non-anesthetized and X-Ray irradiated in the morning with a single exposure at a rate of 1 Gy/min. Since drift in the LD_50/30_ occurs within any laboratory, it is recommended that every laboratory establish a lethality dose-response for each mouse strain at least twice every year [36]. Accordingly, we first exposed different doses of X-ray using PBI model to determine the proper dose in C57BL/6J mice to be used for rhMFG-E8 treatment studies. Our initial experiment determined the LD_50/30_ as 15 Gy, which we then selected to determine the dose response relationship and the optimal dose of the tag-free rhMFG-E8 in treating PBI mice. However, when conducting the dose modification factor studies, 15 Gy had 100% mortality and the LD_50/30_ shifted to 13.7 Gy. We believe this discrepancy is due to the inherent drift that naturally occurs over time [36]. All experiments included contemporaneous vehicle (saline) injection controls. Treatment with 1 μg/kg BW tag-free rhMFG-E8 increased the LD_50/30_ by 1 Gy, strongly supporting further evaluating tag-free rhMFG-E8 as a radiation MCM in large animal models such as dogs or non-human primates. We administered tag-free rhMFG-E8 by subcutaneous injection, as opposed to the intravenous route, to recapitulate a mass casualty scenario during which specially trained caretakers (e.g., nurses) or resources (e.g., materials for phlebotomy) may not be widely available. Furthermore, PBI mice were treated 24 h after radiation exposure, once again reflecting a mass casualty scenario when a large number of victims may overwhelm the system and preclude immediate care. Additional studies are required to determine the efficacy of tag-free rhMFG-E8 for other radiation syndromes such as H-ARS.

To further examine the efficacy of tag-free rhMFG-E8 *in vivo*, an experimental mouse model of AKI was also utilized. Among other causes, AKI can result from any condition associated with decreased renal perfusion including surgery of the aorta, tumor resection, trauma surgery, renal transplantation, and hemorrhagic shock [20]. Ischemia to the kidney typically damages renal tubular epithelial cells by causing profound intracellular ATP depletion, hypoxia and, a rise in intracellular calcium. Although ischemia alone can cause damage to the kidney, subsequent reperfusion occurring during restoration of blood flow results in the production of reactive oxygen species (ROS), damage associated molecular patterns (DAMPs), and other inflammatory mediators, leading to additional cell death. The coexistence of apoptosis and necrosis in renal tissues is a hallmark feature of renal I/R injury. Both types of cell death are implicated in the pathogenesis of AKI which is marked by the loss of tubular epithelial cells and subsequent kidney dysfunction [22, 23]. Amelioration of necrosis and apoptosis can lead to a decrease in inflammation and renoprotection during AKI [21–23]. Indeed, tag-free rhMFG-E8 significantly decreased tubular damage and apoptosis in the kidney cortex at 48 h after AKI. Tag-free rhMFG-E8 also significantly reduced kidney mRNA levels of TNF-α, IL-6 and IL-1β and systemic protein levels of these cytokines, along with improved kidney function biomarkers (i.e., BUN and creatinine) after AKI. We showed protection from AKI after a single dose of tag-free rhMFG-E8 administered immediately after renal I/R, which is a realistic time of administration to prevent AKI associated with high-risk surgical procedures. However, more dosing and time of administration studies are needed to better determine tag-free rhMFG-E8’s therapeutic potential to ameliorate AKI in other settings such as trauma-hemorrhage and septic shock. Nevertheless, our observations clearly show that tag-free rhMFG-E8 is able to reduce renal cell death and attenuate renal inflammation and injury after I/R. Therefore, our data strongly demonstrate that tag-free rhMFG-E8 treatment is renoprotective in AKI.

MFG-E8 was identified as a surface protein of mammary epithelial cells but its physiological role had not been known for a long time. In 2002, Hanayama et al., found that MFG-E8 secreted by macrophages and immature dendritic cells acts as a bridging molecule between apoptotic cells and phagocytes [5, 45]. MFG-E8 has a high affinity to PS which are exposed on apoptotic cells but not with other phospholipids on the cell membrane. While engaged with apoptotic cells, MFG-E8 interacts with phagocytes to stimulate the uptake of apoptotic cells. MFG-E8 contains an RGD motif in the second EGF domain which is a ligand for α_v_β_3_ or α_v_β_5_ integrin expressed on various phagocytes including amateur and professional phagocytes. Although the role of these integrins in apoptotic clearance had been well established, neither of these integrins bind directly to PS. Being able to bind both PS on apoptotic cells and integrins on phagocytes MFG-E8 acts as an opsonin for the uptake of apoptotic cells. MFG-E8 promotes the phagocytosis of apoptotic cells by not only MFG-E8 secreting macrophages but also by other phagocytes. MFG-E8 deficient mice develop inflammation and autoimmunity, including glomerulonephritis, attributed to defects in apoptotic cell removal [7]. In addition to tag-free rhMFG-E8’s potential to be developed as a biological therapeutic for radiation injury and AKI, it might also be developed for other diseases such as atrial fibrosis and atrial fibrillation [46], subarachnoid hemorrhage [47, 48], osteoarthritis [49], and acute pancreatitis [50]. Additionally, MFG-E8 has the potential to become the first example of an entirely new biologic able to promote health and healing by enhancing the removal of dying cells in situations characterized by cytotoxicity, such as infectious diseases, intoxications, envenomations, and in patients undergoing chemotherapy and radiotherapy.

## CONCLUSIONS

In summary, we have successfully generated human cell-expressed tag-free rhMFG-E8 with elevated and stable biological activity and strong safety and pharmacokinetic profiles well suited for the development of therapeutics in acute inflammation and injury. Tag-free rhMFG-E8 effectively ameliorated experimental models of radiation injury and AKI. Therefore, human cell-expressed tag-free rhMFG-E8 is a strong candidate to become an effective radiomitigator as well as a biologic therapeutic for AKI.

## Supplementary Material

1

## Figures and Tables

**Table 1.** Tag-free rhMFG-E8 does not cause acute or chronic toxicity in healthy mice.

Assessment	Baseline	rhMFG-E8 (2 mg/kg), Day 0		
		Day 1	Day 7	Day 28
AST (IU/L)	21.2 ± 4.8	29.3 ± 10.6	13.8 ± 4.0	18.8 ± 0.3
ALT (IU/L)	3.7 ± 1.4	2.7 ± 2.1	2.4 ± 1.3	3.3 ± 1.6
LDH (U/L)	19.0 ± 11.3	52.9 ± 45.9	10.8 ± 5.1	27.6 ± 11.1
Creatinine (mg/dL)	0.3 ± 0.1	0.3 ± 0.1	0.4 ± 0.1	0.4 ± 0.02
BUN (mg/dL)	20.6 ± 2.3	21.8 ± 2.6	20.7 ± 2.2	24.7 ± 0.5
TNF-α (pg/ml)	1.2 ± 0.3	2.0 ± 0.5	1.7 ± 0.3	2.0 ± 0.1
IL-6 (pg/ml)	4.3 ± 0.8	11.4 ± 2.3	6.1 ± 1.1	8.0 ± 1.8

## Data Availability

All data generated or analyzed during this study are included in this published article [and its supplementary information files].

## ABBREVIATIONS

rhMFG-E8: Recombinant human milk fat globule-epidermal grow factor-factor 8
AKI: Acute kidney injury
PBI: Partial body irradiation
DMF: Dose modification factor
EGF: Epidermal growth factor
ARS: Acute radiation syndrome
H-ARS: Hematopoietic acute radiation syndrome
GI-ARS: Gastrointestinal acute radiation syndrome
NV-ARS: Neurovascular acute radiation syndrome
MCM: Medical countermeasure
TBI: Total body irradiation
I/R: ischemia/reperfusion
BUN: Blood urea nitrogen
PS: Phosphatidylserine
PBS: Phosphate buffered saline
HRP: Horseradish peroxidase
TMB: 3,3′,5,5′-Tetramethylbenzidine
ELISA: Enzyme linked immunosorbent assay
ALT: Alanine transaminase
AST: Aspartate transaminase
TNF-α: Tumor necrosis factor-alpha
IL-6: Interleukin-6
IL-1β: Interleukin-1beta
KIM1: Kidney injury marker 1
IL-18: Interleukin-18
TUNEL: Terminal deoxynucleotidyl transferase dUTP nick end labeling
LDH: Lactate dehydrogenase
Gy: Gray
LD_50/30_: Lethal dose
