# Supplementary material for: Development of human cell-expressed tag-free rhMFG-E8 as a radiation mitigator and a therapeutic for acute kidney injury
Source: Res Sq. 2023 May 15:rs.3.rs-2809755. Preprint. [Version 1] doi: 10.21203/rs.3.rs-2809755/v1 (PMC10246299; doi:10.21203/rs.3.rs-2809755/v1)

Supplementary Figure 1. *Tag-free rhMFG-E8 is superior to E coli expressed His-tag rhMFG-E8*. PS binding (**A**) and SVEC4-10 cells adhesion assay (**B**) using tag-free and His-tag rhMFG-E8.

Supplement Figure 1

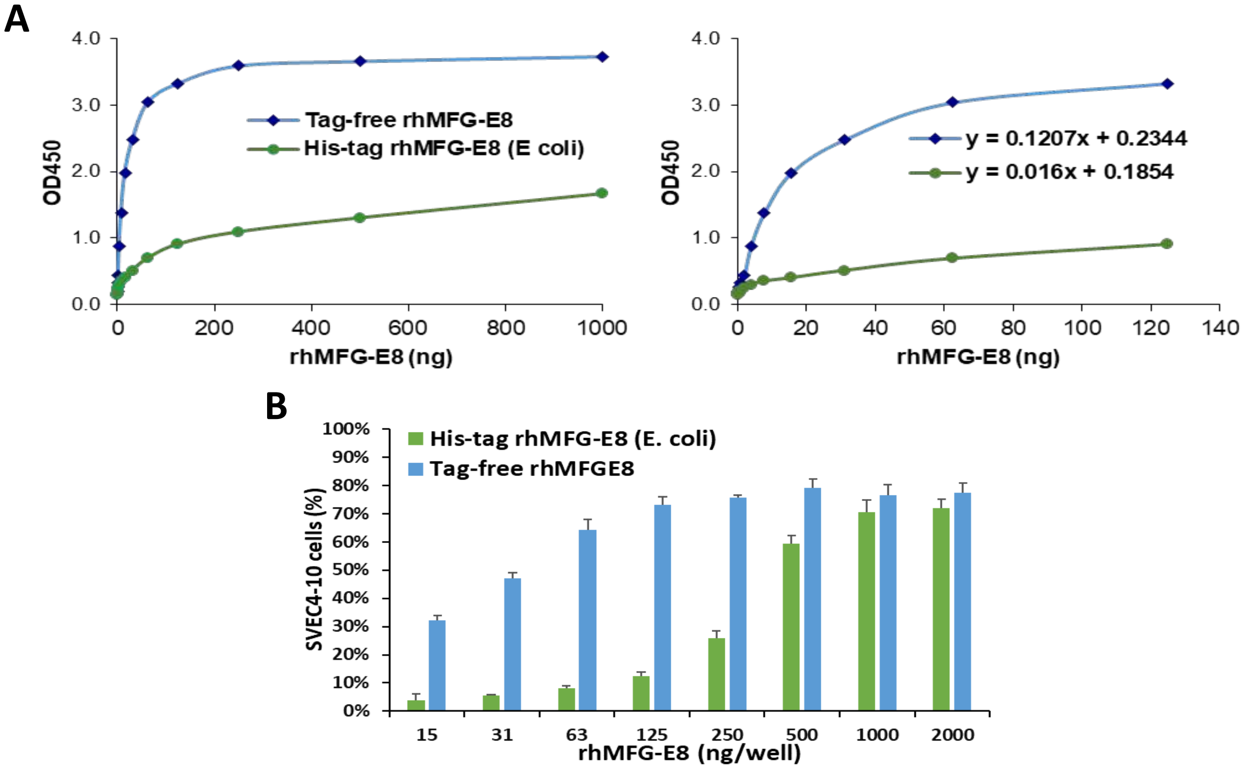

Supplement: 1 [file NIHPPrs2809755v1-supplement-1.pdf]
